# Impact of Pharmacist Counselling on Clozapine Knowledge

**DOI:** 10.1155/2017/6120970

**Published:** 2017-06-13

**Authors:** Ciara Ní Dhubhlaing, Ailish Young, Laura J. Sahm

**Affiliations:** ^1^Pharmacy Department, St. Patrick's University Hospital, James St., Dublin 8, Ireland; ^2^Pharmaceutical Care Research Group, School of Pharmacy, University College Cork, Cavanagh Pharmacy Building, Cork, Ireland; ^3^Department of Pharmacy, Mercy University Hospital, Grenville Place, Cork, Ireland

## Abstract

Clozapine is the only antipsychotic with evidence for efficacy in treatment of resistant schizophrenia but it carries a high side effect burden. Patient information is provided but may be poorly retained. This study aims to examine the impact of pharmacist counselling upon patient knowledge of clozapine. Outpatients, aged 18 years and over, attending St. Patrick's University Hospital, Dublin, participated in this study between June and August 2015. The intervention consisted of pharmacist counselling on two occasions one month apart. Knowledge was assessed using a 28-point checklist devised from the currently available clozapine patient information sources, at baseline and after each counselling session. Ethics approval was obtained. Twenty-five participants (40% female; mean age 45.1 years, SD 9.82; 64% unemployed, 28% smokers) showed an improvement in knowledge scores of clozapine from baseline to postcounselling on each occasion with an overall improvement in knowledge score, from baseline to postcounselling at one month, of 39.43%; *p* < 0.001. This study adds to the evidence that interventions involving pharmacist counselling can improve patient knowledge, whilst the specific knowledge gained relating to recognition of side effects may help patients towards more empowerment regarding their treatment.

## 1. Introduction

Clozapine is an atypical antipsychotic licensed for use in treatment resistant schizophrenia [1]. It is considered to be the gold standard, used after the failure of other drugs due to adverse effects or lack of efficacy [2]. In common with all medicines, it has a range of possible adverse effects, from very rare but possibly life threatening agranulocytosis to the very common troublesome effect of sedation [1]. To manage their medication well and achieve the optimal risk benefit balance, patients should be able to identify these side effects and determine if they may be related to the drug. Pharmacist counselling is a common and broadly accepted method of communicating medication information to patients. A systematic review to determine whether there is evidence to show that pharmacist counselling improves patient knowledge about clozapine amongst outpatient clinic attendees showed a lack of evidence in this area. Five studies [3–7] involving a range of prescribed medications and patient groups were examined. Only one of these was in patients with schizophrenia [5] and none were in those prescribed clozapine exclusively further underpinning the need for more evidence in this area. All studies with one exception [3] demonstrated improvements in patient knowledge of their medication as a result of pharmacist counselling interventions. The exception was a retrospective study where pharmacists had not specifically been tasked with counselling as an intervention. From this review, a single counselling session was noted to result in improvements in knowledge between 8 and 18% overall and where three sessions were undertaken [5] improvements of 43% were achieved. The significance of these improvements in practice is not known and there is evidence that, in the absence of reinforcement, knowledge is lost over time [5, 6].

Limited health literacy (or occurrence of factors associated with limited health literacy) was found to be associated with low knowledge about prescribed medication in all of the reviewed papers. However, the study undertaken in patients with schizophrenia [5] found illness and medication factors to be more strongly correlated with poor medication knowledge than psychosocial or education-related factors; therefore patients with schizophrenia may be a unique cohort when considering medication information needs.

Once discharged from hospital, patients prescribed clozapine attend an outpatient phlebotomy clinic on a regular basis as determined by the product license and the Clozaril Patient Monitoring Service (CPMS) [8] for the duration of their treatment. Despite being prescribed a medication that carries some risk and frequent attendance at hospital clinics, there is no formal structure in place to ensure patients are well informed about their medication on an on-going basis. This research therefore builds on two previous pieces of work (one looking at the appropriateness of clozapine information given to patients and addressing this by developing information leaflets with a higher readability score [9] and another that demonstrated the benefits of pharmacist counselling in improving knowledge about warfarin irrespective of baseline health literacy [6]) in order to identify potential methods of improving patient knowledge and to assess their impact. This research therefore aimed to examine the impact of pharmacist counselling upon patient knowledge of clozapine with the primary objective of determining the change in patient knowledge score after a defined time period and repeated pharmacist counselling. Secondary objectives of assessing baseline health literacy in a cohort of outpatients attending a clozapine clinic using the (Revised) Rapid Estimate of Adult Literacy in Medicine (REALM-R) and thereafter assessing if there is any association between variables of interest and health literacy or the change in patient knowledge were set.

## 2. Methods

### 2.1. Study Design

The study was designed as a prospective interventional study.

### 2.2. Study Setting and Participants

The study participants were outpatients attending St. Patrick's University Hospital (SPUH), Dublin's Clozapine Clinics. St. Patrick's Mental Health Services is Ireland's largest, independent (private), not-for-profit mental health service with a total of 288 adult inpatient beds. There are approximately 2,900 inpatient admissions per year to the hospital accounting for approximately 16% of all admissions to Irish Psychiatric Units and Hospitals in 2012 [10].

### 2.3. Eligibility

All patients attending the Outpatient Clozapine Clinics at SPUH were approached for participation. All those agreeable to take part were included in the study. Data collection was undertaken over an 11-week period from June to August 2015.

### 2.4. Inclusion Criteria

Adult (18 years and older) outpatients attending Outpatient Clozapine Clinics at SPUH who were deemed clinically well enough to participate in the study by their treating team were included in the study.

### 2.5. Exclusion Criteria

Inpatients, those aged less than 18 years, or those unable to provide written informed consent, were excluded.

### 2.6. Confidentiality and Ethical Issues

Ethics approval was obtained from the St. Patrick's Mental Health Services Research Ethics Committee. Prescribers were preinformed by letter to allow time for any objections to inclusion of patients on clinical grounds; however none were received.

Data were anonymized and the principal investigator retained an electronic key separately from the anonymized data on an individually password protected computer. Paper data were stored securely within the pharmacy department, which is a restricted access area. No identifiable results left SPUH premises in paper or electronic format, nor were they made available to any parties other than the principal investigator. All documents and electronic data will be destroyed by shredding and disposal in confidential waste or erasing electronically one year after final publication of the study.

### 2.7. Protocol Procedure Overview

A patient knowledge checklist was devised by a pharmacist from the currently available clozapine patient information sources, that is, (i) the manufacturer's patient information leaflet (PIL) [11] and (ii) the “Choice and Medication” (C&M) PIL (designed for improved usability and readability for patients using mental health services as they are easily understood by most 12 year olds) [12]. This checklist included items which ascertained the patient's knowledge on a range of areas including (i) therapeutic uses of clozapine, (ii) common side effects, and (iii) action to be taken in the event of missed dose. The patient received a maximum of one point per question and a maximum of 28 points overall.

Pilot testing of forms and questionnaires was performed on pharmacy department staff to ensure face validity. Several pharmacists were involved in patient counselling to increase generalizability of pharmacist counselling and reduce the possibility of interviewer bias. Attendees at the weekly Outpatient Clozapine Clinics were approached to participate in this research by a pharmacist. The study was verbally explained and a Participant Information Leaflet was given to the patient to take home. At their next attendance, one month later, participants were asked if they had read the leaflet; if they had any questions; and if they would be interested in participating. If the patient agreed to participate, written and verbal information was again provided on the study and written informed consent obtained.

### 2.8. Assessments Performed

Once enrolled, demographics including age, gender, education level, employment status, smoking status, duration of clozapine treatment, and number of medications coprescribed were collected and health literacy was assessed using the REALM-R [13] health literacy assessment tool.

The patient was asked what forms of information they could recall receiving about clozapine (leaflet, DVD, verbal information, etc.) in the past, and their baseline knowledge of clozapine was then assessed using the 28-point checklist as part of a semistructured interview.

The pharmacist then provided the C&M PIL for the patient to read, counselled the patient on the key information points about clozapine as per the checklist, and addressed any queries or misconceptions arising.

The baseline knowledge checklist was immediately reapplied after counselling, any gaps identified were addressed by the pharmacist, and the time taken for the entire intervention was recorded.

At the next clinic visit (or at 1 month for patients attending more frequently) participants were asked if they had accessed or been given information about clozapine from other sources (e.g., Internet, healthcare professionals, other patients, or friends/family members) in the last month to account for increased knowledge from sources other than pharmacist counselling.

The checklist was reapplied, to ascertain how much of the information was retained. Pharmacist counselling and the provision of the C&M PIL were then repeated and the checklist was applied a final time. The time taken for this repeat intervention was also recorded.

Due to difficulty in recruitment, permission was sought and obtained from the Ethics Committee to conduct interviews by telephone where patients were in agreement.

### 2.9. Sample Size Calculation

The estimated effect size (10.84%) is based upon the Brosnan et al. study [9] in which the average knowledge score for patients (*n* = 40) in the questionnaire before intervention was found to be 8.16 out of 13 and after intervention was found to be 9.57 out of 13 (*p* < 0.01). A sample size of 40 patients was deemed necessary.

### 2.10. Statistical Analysis

The Statistical Package for the Social Sciences (SPSS) Version 20 (IBM Corp.) was used for data analysis. Descriptive statistics include frequencies, percentages, and mean values. Means are reported with standard deviation (SD) where appropriate. Bivariate analyses were conducted to determine any statistically significant relationships between varying parameters, for example, REALM-R score versus employment status. Pearson's correlation coefficient is reported for parametric data and Spearman's rho is used to describe correlations with nonparametric data. Correlations were significant at the 0.05 level unless otherwise specified.

## 3. Results

Twenty-seven patients agreed to participate in the study. There were no drop-outs and no participants were lost to follow-up. One participant did not have time to take part in counselling after the first month and therefore had a two-month gap between interviews whilst another attended the clinic early due to a holiday and therefore had a three-week gap between interviews. The results of these respondents were removed from final analysis.

### 3.1. Patient Demographics

Study participants were typically male, nonsmokers, in their mid-forties, with at least some post-secondary-school education but not in current employment, prescribed approximately five medications, and taking clozapine for 5–10 years as shown in Table 1. All participants identified as “white Irish” ethnicity.

### 3.2. Patient Recall of Clozapine Education Provided Prior to Commencement of Treatment

When asked, 20% of participants could not recall receiving any information about clozapine prior to commencing. The majority of females (90%) recalled receiving at least one form of information, whilst 72% of males recalled receiving clozapine information on initiating treatment.

### 3.3. Knowledge Scores and Changes in Knowledge Scores with Counselling

The mean knowledge score (from a possible maximum score of 28) was low at baseline for the initial counselling session but had more than doubled by the completion of the study as shown in Table 2. The range of scores remained large throughout with the standard deviation remaining relatively consistent.

Despite the statistically significant reduction in knowledge scores in the month between assessments, all patients gained knowledge overall from baseline to completion of the study.

The differences before and after each of the two counselling sessions were statistically significant, as was the mean overall change in knowledge score from the baseline to the final assessment and the drop in scores between counselling sessions (see Table 3).

### 3.4. Knowledge of Adverse Effects of Clozapine

Many patients in this study had good baseline knowledge of the indication, purpose, and frequency of the blood tests required for clozapine. At the conclusion of the study all patients were fully aware of these counselling points and there was an increase in the percentage of respondents having a deeper knowledge of details around this information, for example, what effect clozapine can have on white blood cells and the significance of a “green” blood result. This contrasts with other studies which have reported poor knowledge of the nature and purpose of haematological monitoring with clozapine, with almost half of patients in one study having no understanding of the rationale for the blood tests and less than one in five having adequate understanding [14]. The low scores for knowledge of side effects are also in keeping with the findings of Macpherson et al. [5]

### 3.5. Knowledge of Smoking Interaction Amongst Smokers

In the smokers group, three of the seven participants in the group (42.9%) were unable to identify the clinically significant interaction between tobacco smoke and clozapine prior to counselling and six of the seven participants (85.7%) identified the interaction by the end of the study.

Only two of the eighteen nonsmoking participants (11.1%) were aware of the interaction prior to counselling. At the end of the study, fifteen (83.3%) of these participants correctly identified smoking as having an interaction potential with clozapine. This is of relevance as exposure to tobacco smoke can impact upon clozapine plasma levels in nonsmokers also.

### 3.6. Health Literacy Assessment

The REALM-R assessment of health literacy did not highlight any deficits and it appears that the majority of participants had adequate health literacy. Only one patient would be considered at risk for limited health literacy as per the REALM-R assessment (see Table 4). The knowledge checklist results and the overall change in score from baseline to completion for this participant did not reach statistical significance compared to the mean results for other participants; and the patient's scores remained within one standard deviation of the mean.

### 3.7. Duration of Interview

Interview duration was determined from incomplete data obtained as not all timings were noted. From 14 results obtained for the first interview, the mean time taken was 35.5 minutes, SD 5.6 (range 27–44 minutes). For the second interview, 15 results were recorded and the mean time taken was 22.3 minutes, SD 4.4 (range 16–32 minutes). Counselling was completed in a “real world” context inclusive of general discussion and counselling about nonclozapine medications; therefore interview times spent specifically on clozapine counselling were not clearly definable.

### 3.8. Associations between Variables of Interest and Knowledge

Female patients were prescribed higher numbers of medications (*t* = 2.667, *p* = 0.014); however, there was no statistically significant difference between male and female knowledge scores, and gender was not associated with change in knowledge scores.

There was a moderate negative correlation between age and precounselling scores one month after the first counselling session which was found to be statistically significant (*r* = −0.551, *p* = 0.004) but the difference between older and younger patients after the second counselling session was not statistically significant. Older patients reported accessing information between counselling sessions to a lesser degree than younger patients (*t* = −0.2738, *p* = 0.020) and were prescribed higher numbers of medications (*r* = 0.560, *p* = 0.004).

Education had a moderate positive correlation for the change in knowledge score before and after the first counselling session (rho = 0.459, *p* = 0.021) but no effect on individual knowledge scores or on any other variables. The number of medications prescribed (*r* = −0.557, *p* = 0.004) was moderately negatively correlated with scores one month after the first counselling session. None of the females in this sample were smokers. There was no statistically significant difference between smoker and nonsmoker scores and smoking status did not correlate with change in knowledge scores.

The number of years' participants had been prescribed clozapine did not correlate with any other variables. Patients were asked whether they had accessed any information about clozapine between counselling sessions. Only four patients had done so: two had searched the Internet, one had discussed it with a family member, and one with their doctor. Their scores were not statistically different from that of participants who had not sought further information after the first counselling session. No statistically significant difference was found in the knowledge scores of those interviewed/counselled by telephone and those counselled in person.

Employment status was positively associated with REALM-R (*r* = 0.480, *p* = 0.015) but not with any other variables.

## 4. Discussion

Of the 25 participants in this study, 60% were male. The prevalence of schizophrenia is generally reported to be higher in males than in females and, due to the younger age of onset, males may be overrepresented in first admission schizophrenia studies; however there may be little difference in the lifetime prevalence between males and females [15]. This is similar to the proportion of males attending the clinic as a whole (62%) and also broadly in line with the gender distribution in three of the five studies examined in the literature review [4, 5, 7].

The average age was 45 years. This was largely similar to two studies [5, 7] and somewhat similar to a third [6]. Schizophrenia commonly presents in early adulthood but studies have shown that treatment with clozapine is delayed by an average of four years despite clear guidelines regarding its place in treatment [16]. In this sample, 24% of participants had been taking clozapine for over 10 years and 72% for over 5 years.

Increasing age correlated with lower knowledge scores after the one-month gap of pharmacist counselling but that difference was not significant after the second counselling session. It appears therefore that the drop in scores was compensated for by the conclusion of the second counselling session. A third interview may have been useful to validate this effect. As might be anticipated, increasing age correlated strongly with increased number of medications for comorbidities.

Level of education attained was found to correlate with improvements in the first counselling session but not with the initial postcounselling score alone; that is, the effect of these participants was not large enough to affect the overall initial postcounselling score and the effect of education was not statistically significant thereafter. Only three patients with educational levels less than post-secondary education were part of this sample; therefore further research is required to confirm this effect. Education may be considered as a proxy indicator of health literacy in the absence of specific health literacy assessments [17]. Jarernsiripornkul et al. [7] found higher education to have a significant association with increased knowledge at baseline and after counselling, whilst Macpherson et al. [5] identified a positive association with years of full-time education and knowledge in all groups immediately after counselling and at assessment one month later but, in keeping with results presented here, it did not reach statistical significance.

A positive correlation between employment and health literacy was identified in this study in line with other research [17]. Fewer than 30% of participants in this study were smokers, which is below the average smoking prevalence of 80% in patients with schizophrenia reported in the literature [18]. Patients in our cohort were prescribed a mean of 5.48 (SD 4.49) medications which would indicate polypharmacy. The effect of increasing pill burden upon side effects is well documented as is its effect upon medication adherence [19, 20]. The number of medications prescribed and age were each negatively correlated with the precounselling score after one month. Further study is indicated in order to determine the distinct effects of these variables on knowledge retention. Previous studies [3, 4, 6] have reported age to be strongly associated with knowledge of medication. Macpherson et al. [5] considered the impact of antipsychotic dose on knowledge and reported it to have a statistically significant effect, but Jarernsiripornkul et al. [7] did not find a number of medicines to have a statistically significant impact on knowledge scores. More medications may indicate more severe illness, more comorbidity, and may lead to an increased risk of adverse effects. Interestingly, Macpherson et al.'s [5] results suggest a positive correlation of higher doses with the recall of information and that there is a strong link between medication dose and change in knowledge scores (which they label as educability). Higher doses of psychotropic medications might be expected to be prescribed in more unwell patients and to lead to impairment of memory due to adverse effects such as sedation. In fact, the opposite may be true in practice as effective treatment of schizophrenia may lead to improvements in cognitive functioning [21]. This interaction of medication dose, symptoms, and knowledge score can also be expected to change over time as the patients' dose is titrated, or as symptoms change with age or external effects.

Whilst it is estimated that 40% of Irish adults have limited health literacy [22], only 4% of this sample population were found to be at risk for poor health literacy. The REALM-R was chosen for its brevity given the extent of the engagement required of the participant in the study. Although the REALM-R has been correlated with the 66-item REALM [13], neither has been validated in a population of patients with schizophrenia. The large proportion of participants with higher levels of education is also likely to have affected the REALM-R score.

In order to assess the health literacy of this small sample, the bar for REALM-R score was set at seven out of a maximum of eight. With this adjustment, a higher REALM-R score was associated with a higher baseline score; however, the result was only marginally significant. There was no association with change in knowledge score. Collins et al. [6] found REALM score to be strongly correlated with higher baseline knowledge score but, as is suggested in this study, not with improvements in score after counselling. Schmitt et al. [3] found adequate to marginal health literacy or “at least some college education” to be strongly correlated with knowledge in their retrospective cross-sectional study. A larger sample would be advisable in order to draw conclusions directly from the health literacy scores obtained in this study.

The difference between the mean increases after each pharmacist counselling session was statistically significant; therefore it can be said that repeated counselling demonstrated benefits over a single counselling intervention.

Controlling for all other variables, the effect of initial baseline results on total improvement was significant; that is, the higher the initial baseline score, the less the improvement attained overall. Consequently, the participants who scored lower at the initial baseline assessment attained greater increases by the end of the study. This is not unexpected, as lower scoring participants had greater scope for gaining knowledge but it also indicates that those with least knowledge at baseline may have the most to gain from pharmacist counselling. Other studies reviewed did not comment on whether this was the case in their results but Macpherson et al. [5] did identify a cohort of patients that scored minimally at baseline and follow-up and suggest that there may be subgroup unable to benefit from education. In this study two pharmacist counselling sessions led to sustained higher scores supporting their [5] finding against using a single explanatory session in favour of more prolonged education. Macpherson et al. [5] also contend that “intermittent booster education sessions are likely to consolidate learning” which is somewhat supported by the findings of this study; however longitudinal studies are indicated to confirm these conclusions. Contrary to their findings in patients with schizophrenia where a subgroup of patients scored minimally at baseline and follow-up which the authors attribute to the complex “relationships between cognition, educability, and schizophrenic symptomology” [5], this study found that all patients benefited from counselling. Although the benefit was not uniformly distributed, pharmacist counselling was shown to be beneficial for knowledge gain in all participants. Many patients did report trouble with memory and found it hard to maintain concentration for the course of the intervention but the results demonstrate there are benefits even for patients shown to be at increased risk for low knowledge (i.e., older patients who are prescribed more medications).

### 4.1. Limitations

Twenty-seven patients from a total of 82 clinic attendees (32.9%) agreed to participate in the study. Two participants were excluded from the results as the gap between counselling sessions was more or less than one month. Larger numbers would be desirable in order to increase the generalizability of the study results.

Counselling sessions consisted of general discussions about mental health and nonclozapine medication queries as well as the counselling intervention of interest. It was therefore difficult to ascertain the specific clozapine counselling intervention time and to determine the cost-benefits of pharmacist time spent on clozapine counselling. However, the naturalistic counselling methods make the outcomes more applicable to “real world” pharmacist counselling. The involvement of three pharmacists in the counselling also improves the generalizability of this intervention.

## 5. Conclusion

All participants in this study gained knowledge of clozapine as a result of pharmacist counselling, which adds to the evidence that pharmacist counselling can improve patient knowledge of medication in a broad range of patient groups.

This study also demonstrates that a proportion of the knowledge gained was preserved over time and that reinforcement of information leads to further gains.

Health literacy and illness factors may impact upon patient knowledge and should be considered when counselling patients, particularly when providing written information. This study provides some evidence that improvements in knowledge are not limited by baseline health literacy; however further research is needed to confirm this view.

## Figures and Tables

**Table 1:** Study participant demographics.

Study participant demographics	*n* =	%
*Gender*		
Female	10	40
Male	15	60
*Age (years)*		
Mean (standard deviation)	45.08 (9.82)	
Range	32–63	
*Level of education obtained*		
Primary school	2	8
Junior certificate	1	4
Leaving certificate	—	—
College/further education	7	28
University undergraduate degree	8	32
University postgraduate qualification	3	12
Master's degree	4	16
*Employed*		
Full time	3	12
Part time	6	24
No	16	64
*Smokers*	7	28
*Years on clozapine*		
<0.5	1	4
0.5–1	—	—
1–5	6	24
5–10	12	48
>10	6	24
*Number of prescribed medications*		
Mean (standard deviation)	5.48 (4.49)	
Range	1–18	

**Table 2:** Mean pre- and postcounselling knowledge scores (*n* = 25).

*First counselling session*	
*Baseline knowledge score (T1)*	
Mean score	9.16
Range	2–17
Standard deviation	4.53
*First postcounselling score (T2)*	
Mean score	17.22
Range	5–26
Standard deviation	5.41

*Second counselling session*	
*Knowledge score 1 month after counselling (T3)*	
Mean score	14.36
Range	2–22
Standard deviation	5.27
*Second postcounselling score (T4)*	
Mean score	20.24
Range	11–27
Standard deviation	4.48

T1 = baseline knowledge score; T2 = first postcounselling knowledge score; T3 = knowledge score 1 month after counselling; T4 = second postcounselling knowledge score.

**Table 3:** Changes in knowledge scores.

		Change in score (as a % of max. score)	Test of statistical significance
*Change in knowledge score after first counselling session (T2-T1)*			
Mean change	8.00	28.57%	*t* = 8.69, *p* < 0.001
Range	0–17		
Standard deviation	4.60		
*Change in knowledge score one month after first counselling session (T3-T2)*			
Mean change	−2.80	−10.00%	*t* = 2.99, *p* = 0.006
Range	−13–4		
Standard deviation	4.68		
*Change in knowledge score after second counselling session (T4-T3)*			
Mean change	5.84	20.86%	*t* = 8.92, *p* < 0.001
Range	1–14		
Standard deviation	3.28		
*Overall change in knowledge score (T4-T1)*			
Mean change	11.04	39.43%	*t* = 13.10, *p* < 0.001
Range	5–22		
Standard deviation	4.25		

T1 = baseline knowledge score; T2 = first postcounselling knowledge score; T3 = knowledge score 1 month after counselling; T4 = second postcounselling knowledge score.

**Table 4:** REALM-R health literacy assessment scores.

REALM-R score	*n* =	%
6	1	4
7	7	28
8 (maximum)	17	68

A score of 6 or less should be considered to be at risk for poor health literacy.
